# Application of metagenomic next-generation sequencing in the diagnosis of infectious diseases

**DOI:** 10.3389/fcimb.2024.1458316

**Published:** 2024-11-15

**Authors:** Yu Zhao, Wenhui Zhang, Xin Zhang

**Affiliations:** ^1^ Department of Urology Surgery, Beijing Chao-Yang Hospital Affiliated to Capital Medical University, Beijing, China; ^2^ Department of Hepatobiliary Surgery, Beijing Chao-Yang Hospital Affiliated to Capital Medical University, Beijing, China

**Keywords:** metagenomics next-generation sequencing (mNGS), infectious disease, pathogens, microorganisms, diagnosis

## Abstract

Metagenomic next-generation sequencing (mNGS) is a transformative approach in the diagnosis of infectious diseases, utilizing unbiased high-throughput sequencing to directly detect and characterize microbial genomes from clinical samples. This review comprehensively outlines the fundamental principles, sequencing workflow, and platforms utilized in mNGS technology. The methodological backbone involves shotgun sequencing of total nucleic acids extracted from diverse sample types, enabling simultaneous detection of bacteria, viruses, fungi, and parasites without prior knowledge of the infectious agent. Key advantages of mNGS include its capability to identify rare, novel, or unculturable pathogens, providing a more comprehensive view of microbial communities compared to traditional culture-based methods. Despite these strengths, challenges such as data analysis complexity, high cost, and the need for optimized sample preparation protocols remain significant hurdles. The application of mNGS across various systemic infections highlights its clinical utility. Case studies discussed in this review illustrate its efficacy in diagnosing respiratory tract infections, bloodstream infections, central nervous system infections, gastrointestinal infections, and others. By rapidly identifying pathogens and their genomic characteristics, mNGS facilitates timely and targeted therapeutic interventions, thereby improving patient outcomes and infection control measures. Looking ahead, the future of mNGS in infectious disease diagnostics appears promising. Advances in bioinformatics tools and sequencing technologies are anticipated to streamline data analysis, enhance sensitivity and specificity, and reduce turnaround times. Integration with clinical decision support systems promises to further optimize mNGS utilization in routine clinical practice. In conclusion, mNGS represents a paradigm shift in the field of infectious disease diagnostics, offering unparalleled insights into microbial diversity and pathogenesis. While challenges persist, ongoing technological advancements hold immense potential to consolidate mNGS as a pivotal tool in the armamentarium of modern medicine, empowering clinicians with precise, rapid, and comprehensive pathogen detection capabilities.

## 1 Introduction

Infectious diseases, a collective term for diseases caused by pathogenic microorganisms, remain a major threat to global public health, and traditional pathogenetic diagnostic methods no longer meet the needs of clinical diagnosis and treatment (Smiley Evans et al., 2020). Rapid identification of pathogens from infected body fluid compartments is essential, as empirical antimicrobial therapy is often suboptimal, leading to increased morbidity and mortality (Singal et al., 2014; Glimåker et al., 2015; Lucas et al., 2016; Costales and Butler-Wu, 2018). In patients with severe infections, early detection of the causative microorganism is essential for early clinical interventions to be instituted and appropriate antimicrobials to be administered (Kumar et al., 2009; Liesenfeld et al., 2014; Barlam et al., 2016; Messacar et al., 2017). However, timely and accurate diagnosis remains extremely challenging for many patients. Many common pathogens are difficult or impossible to culture *in vitro*, deep infections often require invasive biopsies of infected tissues for diagnosis, and the use of broad-spectrum antibiotics prior to pathogen identification often confounds the specific diagnosis, leading to more effective and less toxic antimicrobial therapy (Fenollar and Raoult, 2007; Fishman, 2007; Tomblyn et al., 2009; Mancini et al., 2010; Paul et al., 2010; Kumar, 2014; Barlam et al., 2016). Previous studies have shown that a significant proportion of unknown pathogens are present in severe pneumonia, bacteremia, eye infections and central nervous system (CNS) infections (Li et al., 2018; Blauwkamp et al., 2019; Wilson et al., 2019). Metagenomic next-generation sequencing (mNGS) is useful when conventional microbiological tests fail to identify infection in suspected cases. It is capable of simultaneously detecting virtually all known pathogens from clinical samples (Simner et al., 2018; Chiu and Miller, 2019; Gu et al., 2019). Compared with traditional pathogenic diagnostic methods (culture, mass spectrometry, immune-associated antigen-antibody detection and nucleic acid detection technology, etc.), mNGS is a non-targeted, broad-spectrum pathogenicity screening technology, which has been developed rapidly in recent years and has been widely used in the precise diagnosis of infectious disease pathogenic microorganisms, especially in the diagnosis of infections caused by critical, difficult, rare, and new-emerging pathogens. Therefore, this paper introduces the basic principles and sequencing platform of mNGS, evaluates its strengths and weaknesses, summarizes its applications in various organ system infections, and finally looks forward to the future development.

## 2 Overview of mNGS

mNGS is a next-generation macro-genome-based sequencing technology that enables rapid sequencing of nucleic acids in samples (human and pathogenic microorganisms) and compare them to human genome sequences and pathogenic microbial genome sequences to learn the species and proportions of microorganisms in the sample. It is a technique to obtain nucleic acid sequences from samples (human and pathogenic microorganisms) by rapid sequencing on a second-generation sequencing platform, and compare them with human genome sequence libraries and pathogenic microorganism genome sequence libraries to know the types and proportions of microorganisms in the samples (Yi et al., 2024). It provided an ideal approach for genomic analysis of all microorganisms in a sample, not just those suitable for culture (Wooley et al., 2010).

### 2.1 Technical principle

**Figure 1:**
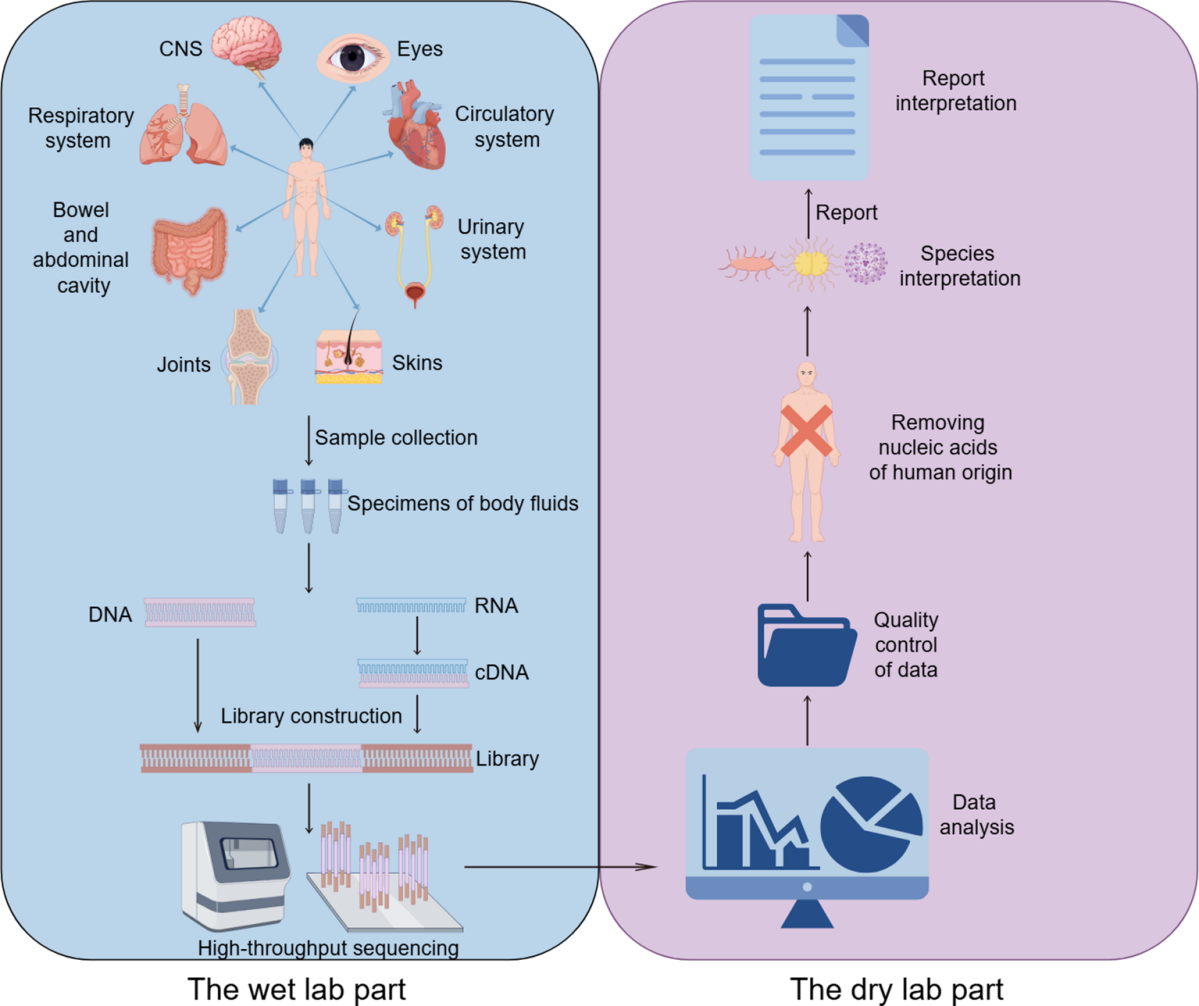
mNGS workflow in clinical application. CNS, Central nervous system.

The process of high-throughput sequencing of pathogens consists of two main parts (Gu et al., 2019): the wet lab part (laboratory testing) and the dry lab part (bioinformatic analysis). The wet lab part includes sample collection, nucleic acid extraction, library construction and high-throughput sequencing. The dry experimental part includes quality control of data, removal of human sequences, sequence comparison of microbial species sequences, and analysis of drug resistance or virulence genes (**Figure 1**).

mNGS is a NGS assay allowing for comprehensive detection of all genes in all organisms in a given sample (Liu et al., 2024). It can be used for bacterial, fungal, parasitic, and various viral infections and is primarily a sequencing comparison process for nucleic acids extracted from infected samples. Because of the different processes for targeting deoxyribonucleic acid (DNA) and ribonucleic acid (RNA) in nucleic acids, an assessment should be made as to which method of testing to use before finalizing the test. DNA testing is recommended when infection by pathogens whose nucleic acids are DNA, such as bacteria, fungi, DNA viruses, parasites, etc., is suspected; RNA testing is recommended if RNA viral infection is suspected; and co-testing of DNA and RNA is recommended if it is not possible to determine which type of viral infection is involved. In addition, the diagnosis of infectious diseases requires that specific samples must first be collected from the site of primary infection before the samples can be preprocessed (Gu et al., 2019). For example, bronchoalveolar lavage fluid (BALF) and sputum are typically recommended for lung infections, while cerebrospinal fluid (CSF) is recommended for CNS infections. While library construction, sequencing, and bioinformatics analysis are the same for different samples, pretreatment and nucleic acid extraction vary depending on the sample source.

### 2.2 Sequencing platforms

The most commonly used mNGS sequencing platform is the Illumina platform, which is based on the core principle of sequencing by synthesis (SBS), which consists of four main steps: DNA library construction, BALF fluid Flowcell adsorption, bridge polymerase chain reaction (PCR) amplification, and SBS (Bentley et al., 2008). Illumina’s sequencing principle of adding only one deoxy-ribonucleoside triphosphate (dNTP) at a time makes it possible to solve the problem of inaccurate sequencing due to the polymerization of identical bases (e.g., when the DNA strand contains repetitive sequences such as AAAAAAA). The sequencing principle of adding only one dNTP at a time makes Illumina a good solution to the problem of inaccurate sequencing due to the polymerization of the same base (e.g., when the DNA strand contains repetitive sequences such as AAAAAA, most sequencing platforms are prone to errors of over-reading or under-reading one base). Currently, Illumina sequencing has an error rate as low as 0.1% (e.g., HiSeq series), with base substitutions being the main source of error.

The Thermo Fisher Ion Torrent next-generation sequencing (NGS) platform is based on the principle of hydrogen ion semiconductor sequencing for non-destructive high-throughput sequencing of nucleic acid fragments. Using natural bases without any artificial modification during the synthetic extension of the nucleic acid chain, the ATGC base biosignal of the nucleic acid fragment to be tested is quickly and accurately converted into digital information by semiconductor technology. Without the need for complex, expensive and environmentally demanding optical detection and scanning imaging systems, and without the use of artificially modified bases, the Ion Torrent platform is more cost-effective, smaller, and faster than other sequencing technologies, completing the sequencing of a single 200 bp sequence in 2 to 2.5 hours.

In 2016, Beijing genomics institute (BGI) announced the BGISEQ-500 sequencing platform, which has a general NGS workflow and stepwise sequencing program similar to that of the Illumina series; however, the two templates are distinctly different (Goodwin et al., 2016). The follow-on DNA nanospheres technology in the BGISEQ-500 platform, which is specifically used for library preparation, is different from the library construction protocol used in the Illumina series (Drmanac et al., 2010). the BGISEQ-500 utilizes both single-end (SE) and paired-end (PE) modes, comparable to the latest Illumina model, the HiSeq4000. the BGISEQ-500 has published relatively high throughput data, and may be suitable for high throughput transcriptome studies.

Nanopore sequencing permits the inclusion of bacteria and fungi with marker genes of different sizes in the same sequencing library by detecting the electrical signals of DNA/RNA as it passes through nanopore proteins (Han et al., 2024). Although nanopore metagenome sequencing based on real-time analytical pathways reduces the detection time to less than 6 hours, it still faces challenges such as insufficient sensitivity, high sequencing errors, and elevated detection costs.

In 2021, the Association of Biomolecular Resource Facilities (ARBF) led an ABRF NGS Phase II study published in Nature Biotechnology, based on multiple sequencing platforms from Illumina, Pacific Biosciences, Thermo Fisher Scientific, Oxford Nanopore Technologies, and Genapsys, The team sequenced the same human genome family, three individual strains, and a mixture of ten bacterial metagenomes in multiple laboratories based on multiple sequencing platforms from Illumina, Pacific Biosciences, Thermo Fisher Scientific, BGI, Oxford Nanopore Technologies, and Genapsys. The data from each platform were compared in a comprehensive and systematic way to analyze the performance differences and sequencing quality of each sequencing platform. The data show that among the short-read-long sequencing platforms, Illumina’s HiSeq 4000 and HiSeq X10 platforms provide the most consistent and highest genome coverage, while BGI’s BGISEQ-500 and MGISEQ-2000 platforms provide the lowest sequencing error rate. Among the long read-length sequencing platforms, the PacBio CCS has the highest reference-based mapping rate and the lowest non-mapping rate. Both the PacBio CCS and Oxford Nanopore’s PromethION, MinION platforms show the best sequence localization performance in both repeat sequence-rich regions and across homopolymer assays. The NovaSeq 6000 uses the 2×250 bp read chemistry is the most powerful instrument for capturing known insertion and deletion (INDEL) events.

## 3 Advantages and limitations of mNGS in clinical applications

### 3.1 Advantages of mNGS in clinical applications

mNGS is increasingly recognized for its groundbreaking capabilities in the field of infectious disease diagnostics. One of the principal advantages is its comprehensive and unbiased approach; it does not require prior hypotheses about which pathogens might be present. This allows for the simultaneous detection and identification of a wide array of pathogens—including bacteria, viruses, fungi, and parasites——from a single sample (Rodino and Simner, 2024). Firstly, it can identify nearly any pathogen present in a sample without needing specific probes or primers for each one. This is particularly beneficial for detecting rare pathogens, those presenting atypically, or those for which no targeted diagnostics exist. Secondly, it is especially useful for diagnosing infections in immunocompromised patients, where the range of possible infecting organisms is broader and often includes lower common pathogens. Thirdly, it can detect genes responsible for resistance to antimicrobials, providing crucial information for guiding treatment decisions. Besides, Ithas the advantage of timeliness compared to culture methods, which typically take 2 - 3 days to obtain results, and even more than a week for fussy bacteria, such as mycobacterium tuberculosis (MTB) (Mu et al., 2021). The average turnaround time for conventional mNGS is 48 hours (Han et al., 2019; Gu et al., 2021). One study reported a turnaround time of only 6 hours for the detection of pathogenic microorganisms using the mNGS technology on a nanopore platform (Gu et al., 2021; Mu et al., 2021). Finally, It is also highly effective in situations where patients have already been treated with antibiotics, which can inhibit pathogen growth in cultures and lead to negative results despite ongoing infection (Zhang et al., 2019). In summary, mNGS can provide comprehensive and rapid results that can guide clinicians to more precise and effective treatments.

### 3.2 Limitations of mNGS in clinical applications

First, mNGS is unable to determine whether the sequences it detects are from live or dead pathogens, so it still does not solve the perennial problem of identifying colonizing and pathogenic pathogens. Thus, the detection of DNA only indicates what organisms are present, not whether they are biologically active, and even blood specimens is still unable to differentiate pathogenic bacteria from transient bacteremia and from microbial nucleic acid fragments contained in leukocytes. Perhaps the detection of RNA would help in this regard, as the presence of RNA could indicate that the organism is transcriptionally active (Liu et al., 2024). As a result, mNGS cannot serve as first line diagnostic assay due to its low sensitivity (Liu, 2024; Liu et al., 2024).

Second, it is limited to roughly determining the species of pathogenic microorganism and estimating the approximate proportion of microorganisms (quantifying pathogen reads as a percentage of the total number of sequences reads). If the pathogenic microorganism is a particularly small proportion of the genus, it is highly unlikely to produce a negative result. Therefore, a negative result may simply reflect a sample with a high non-microbial nucleic acid component (denominator) and/or a low microbial nucleic acid component (molecule), rather than a lack of pathogens (Liu et al., 2024).

Third, some low levels of intracellular bacteria, e.g., MTB, Legionella, Brucella, and fungi with thick cell walls will be detected at lower rates (the latter require special treatment to disrupt the cell wall and expose the DNA). Therefore, for all types of pathogens, nucleic acid recoveries may not be equal under the same DNA extraction technique. Therefore, different extraction methods should be used to detect specimens of different target microorganisms.

Fourth, there are no standardized procedures and standards to avoid contamination of nucleic acids in the steps from specimen collection to processing and the environment, so that the results of different laboratories tend to be similar.

Fifth, the relatively short reading sequence (300 bp) of mNGS makes it difficult to obtain the full-length sequence of drug resistance genes, and it is not possible to correlate the drug resistance genes with the corresponding microbial species. The length of three-generation sequencing can reach 1200bp, which can potentially cover the full length of drug-resistant genes, and is a good help for the determination of drug-resistant genes.

Sixth, the positive controls should cover the range of microorganisms likely to be encountered in blood samples, such as: enveloped and non-enveloped viruses, RNA and DNA genomes, Gram-positive and negative bacteria, mycobacteria and parasites. However, we do not know in advance what pathogens are present in the specimen to be tested. Negative controls are equally problematic because the water and sample matrix do not adequately reflect the background present in normal healthy blood (other) specimens.

Last but not least, interpreting the results generated by the sequencing lab is one of the biggest headaches in mNGS clinical practice today. Some institutions have implemented precision medicine teams-composed of microbiology, computational biology, infectious disease, and other clinicians-to discuss results and provide interpretation of results prior to reporting. This may be the best option at this time. Furthermore, the expensive pricelimits its widespread clinical use.

### 3.3 Comparison between mNGS and targeted NGS

tNGS is a targeted high-throughput sequencing technology for specific genes or genomes. tNGS, unlike mNGS, performs high-throughput sequencing of only specific gene sequences, thereby increasing detection sensitivity while eliminating interference from host nucleic acids (Yi et al., 2024). tNGS is primarily designed for the detection of dozens to hundreds of known pathogenic microorganisms and their drug-resistant genes in a sample. Depending on the enrichment strategy, there are two main technical routes for tNGS enrichment: one is PCR amplicon enrichment, i.e., enrichment of small viral genomes by PCR amplification of viral genomes with hundreds to thousands of base pairs using primers complementary to known nucleotide sequences before NGS sequencing; Another type of enrichment is hybridization-targeted probe enrichment, i.e., small RNA/DNA probes that are usually first designed to be complementary to the pathogen reference sequence (Pham et al., 2023; Chen et al., 2024; Yi et al., 2024). Unlike methods based on specific PCR amplicons, probe-targeted enrichment allows the entire genome to be covered by overlapping probes that are used in a hybridization reaction to capture complementary DNA sequences that bind to their sequences (Chen et al., 2024). Thus, tNGS combines the advantages of PCR and NGS.

A study conducted in 2003 on the molecular diagnosis of infective endocarditis by PCR amplification and direct sequencing of valvular tissue DNA can be considered a prototype of tNGS. Its results showed significant concordance between tNGS results and histopathologic evaluation, with concordance rates as high as 93.1% (27/29) for positive samples and 92.9% (13/14) for negative samples (Gauduchon et al., 2003). Several subsequent studies have validated this finding (Marín et al., 2007; Vondracek et al., 2011; Maneg et al., 2016). However, relying solely on tNGS for clinical testing is not advisable because of its occasionally limited predictive power for negative samples (Maneg et al., 2016). In the challenge posed by the COVID-19 pandemic, tNGS has been used for infectious disease surveillance and genotyping (Cheng et al., 2023; Ramos et al., 2023). In addition, Chao et al. reported the use of tNGS for pathogen identification in patients with acute lower respiratory tract infections. The positive rate of tNGS was as high as 95.6% based on the gold standard sputum culture (Chao et al., 2020). Recent studies have also reported the use of tNGS in the identification of rare pathogens, including Legionella pneumophila, Chlamydia psittaci, Whipple’s bacillus, Aspergillus fumigatus, and Cryptococcus neoformans (Du and Chen, 2023; Li et al., 2023; Ren et al., 2023; Zhang et al., 2023). To date, the value of tNGS has been demonstrated for clinical applications in the areas of bloodstream infections, central nervous system infections, and tuberculosis (Cabibbe et al., 2020; Deng et al., 2020; Mensah et al., 2020; Jouet et al., 2021; Mesfin et al., 2021; Chen et al., 2022; Kunasol et al., 2022; Sibandze et al., 2022; Yang et al., 2022; Jiang et al., 2023). tNGS cannot be run on its own, but is used in conjunction with conventional assays. This approach may contribute to an effective and accurate clinical diagnosis. By combining the ubiquity of conventional testing with the high specificity and sensitivity of tNGS, clinicians are better able to make a more accurate diagnosis. This integrated diagnostic strategy may improve patient prognosis through timely and appropriate therapeutic interventions. In conclusion, tNGS may have a role in the diagnosis of infectious diseases. By addressing the limitations of current assays, tNGS could provide a more refined, accurate and comprehensive approach to pathogen detection (Chen et al., 2024). In summary, tNGS may bridge the diagnostic gap between traditional assays and mNGS.

There are significant differences between mNGS and tNGS (**Table 1**). Firstly, tNGS sequences only specific regions or specific genes, usually for known genes, pathogens, or specific genomic loci, i.e., this method requires the target sequence to be set before the experiment begins. In contrast, mNGS sequencing is wide-ranging and can sequence all DNA/RNA fragments in a sample without bias. This means it can recognize all genomic information in the sample, including pathogens, human genome, microbiota, etc. Therefore, tNGS possesses greater sensitivity and specificity but poor flexibility. Secondly, tNGS is usually used for the detection of known targets, such as gene mutation, genetic disease related gene detection, cancer gene detection, etc. It is very effective for rapid and precise detection of known pathogens, and is suitable for diagnosing specific diseases or for personalized medicine. However, mNGS is suitable for identification of unknown pathogens, analysis of complex microbial communities, detection of infectious diseases, and screening of drug resistance genes. Due to its extensive sequencing capabilities, mNGS can be used clinically to discover novel pathogens or complex sources of infection. Thirdly, the results of tNGS are more direct and precise due to clear targeting, relatively small amount of data, and simple analysis process. Its data processing is faster and suitable for rapid response in clinical diagnosis. On the other hand, mNGS is more complicated to analyze due to the large amount of data generated and the inclusion of a large amount of irrelevant information (e.g., host genes, environmental strays, etc.), which requires powerful bioinformatics tools to filter and interpret the data, and the process of data processing and analysis is time-consuming and the results may have uncertainties. Finally, tNGS is less costly and faster to analyze, making it suitable for diagnostics or research with specific targets. In contrast, mNGS, due to its high coverage, is more costly and relatively time-consuming, and is suitable for complex, unresolved infection cases or studies that require extensive exploration. In summary, tNGS is a targeted, low-cost sequencing method for rapid and accurate detection of known targets, while mNGS is a broad, unbiased sequencing method for identification of unknown pathogens and analysis of complex environments.

**Table 1:** The comparison between mNGS and tNGS.

	mNGS	tNGS
**Methodology**	1 Direct extraction of DNA/RNA 2 Without predefining specific pathogens 3 High-throughput sequencing	1 Targeted enrichment and ultra-multiplex PCR 2 With predefining specific pathogens 3 High-throughput sequencing
**Sequencing Scope**	Wide	Narrow
**Sample Size**	Small	Small
**Data Volume**	Large	Small
**Analysis Complexity**	Sophisticated	Simpler
**Sensitivity**	Normal	High
**Specificity**	Normal	High
**Cost and Time**	High and long	Low and short
**Flexibility**	Flexible	Inflexible
**Clinical Application Time**	Short	Short
**Application**	Unknown pathogens: 1 Analysis of complex microbial communities 2 Detection of infectious diseases 3 Screening for drug resistance genes	Known pathogens: 1 Genetic mutation 2 Genetic testing for genetic diseases 3 Cancer genetic testing
**Targeting pathogens**	Bacteria, viruses, mycoplasma, etc.	Bacteria, viruses, fungi, mycoplasma, etc.

mNGS, Metagenomic next-generation sequencing; tNGS, Targeted metagenomic next-generation sequencing; DNA, Deoxyribonucleic acid; RNA, Ribonucleic acid; PCR, Polymerase chain reaction.

In addition to tNGS, there are a variety of microbiological testing methods available, each with its own advantages and disadvantages, as shown in **Table 2**. In conclusion, mNGS is not currently a replacement for current conventional microbiological testing methods, but should be viewed as a complement to these traditional methods.

**Table 2:** Comparison of testing methods for diagnosing infectious diseases.

Diagnostic test	Advantages	Disadvantages
**Serology**	1 Low cost 2 Suitable for acute infections	1 False-negatives 2 False-positives
**Culture**	1 Able to accommodate large sample volumes 2 Low cost 3 Wide range of applications	1 Sensitivity is limited by antibiotics and antifungal drugs 2 Sensitivity limited by picky microorganisms 3 Limited use in viral assays 4 Long time to produce results, especially in antacid and fungal cultures
**Microscopy and staining (eg, Gram stain, auramine–rhodamine, calcofluor-white)**	1 Rapid 2 Low cost	1 Low sensitivity 2 Low specificity
**Matrix-assisted laser desorption/ionization time of-flight mass spectrometry**	1 High specificity 2 Rapid after culture	1 Requires culture-positive isolate
**Direct PCR**	1 Simple 2 Rapid 3 Low cost 4 Potential for quantitative PCR	1 Dependent on assumptions 2 Primers are not always effective 3 Limited to a small part of the genome
**Multiplex PCR**	1 Fast 2 Detect a wide range of microorganisms	1 Low specificity 2 False positives
**Targeted universal multiplex PCR (eg, 16S, ITS) for Sanger sequencing**	1 Ability to distinguish multiple species within a pathogen type	1 Primers are not always effective 2 Limited to a small part of the genome
**Targeted universal** **multiplex PCR (eg, 16S, ITS) for NGS**	1Ability to distinguish multiple species within a pathogen type 2 Multiplexing capability 3 Potential for quantitation	1 Primers are not always effective 2 Expensive 3 time consuming 4 Often requires more than one amplification 5 Limited to a small part of the genome
**Amplicon sequencing**	1 Bacteria, fungi 2 Low expenditure 3 Low biomass required, no host contamination 4 Low volume of data generated, easy to analyze 5 Reliable database	1 Not applicable to viruses 2 Low species resolution 3 Functional genes not available 4 Low biomass may lead to false negatives 5 Results of community diversity analyses varied across variable regions
**Targeted NGS**	1 Suitable for initial screening of hospitalized patients 2 Unaffected by human genome and background flora 3 High ability to detect engulfed pathogens 4 High ability to detect drug resistance and virulence 5 Ability to add new targets according to clinical needs	1 Short clinical application time 2 Inability to recognize new pathogens 3 Incomplete database
**mNGS**	1 Identify viruses, fungi, archaea and protozoa 2 Timeliness 3 Without needing specific probes or primers for each one 4 It is especially useful for diagnosing infections in immunocompromised patients 5 It can detect genes responsible for resistance to antimicrobials 6 It applies when you are already receiving antibiotics	1 High DNA quality requirement 2 Host contamination 3 Not easy to assembly and complex analysis process 4 False positive results 5 Expensive
**2bRAD-M**	1 High technical reproducibility 2 High species resolution 3 It can be used for low biomass, heavily degraded samples, high host contamination samples 4 Simultaneous detection of bacteria, fungi and archaea 5 Host snp analysis, human genetic analysis 6 Microbial diversity analysis and host SNP analysis can be combined for GWAS analysis	1 Expensive 2 It cannot detect small and short genes, such as viruses 3 It can do GWAS analysis, but the number of snp is low Cannot recognize new pathogens
**MobiMicrobe**	1 Reliable genomes 2 High quality genome assembly 3 Precise genomic analysis at the strain level to discover new uncultured strains 4 Mining inter-strain relationships to analyze horizontal gene transfer 5 Single-cell level of host-phage binding	1 Low genome coverage of Gram-negative bacteria

PCR, Polymerase chain reaction; ITS, Internal transcribed spacer sequencing; NGS, Next-generation sequencing; mNGS, Metagenomic next-generation sequencing; DNA, Deoxyribonucleic acid; SNP, Single nucleotide polymorphism; GWAS, Genome-wide association study.

## 4 Application of mNGS in infections in different organ systems

### 4.1 Bloodstream Infections

The composition of causative organisms varies from sepsis to sepsis; in recent years there has been an increase in the number of cases of gram-negative, anaerobic and fungal sepsis, but gram-positive organisms remain the most common (Zhou et al., 2016). There is also a subset of culture-negative sepsis patients for whom the causative organism remains undetermined. In patients with severe sepsis, failure to diagnose the pathogen in a timely manner can lead to receiving inappropriate and mismatched antimicrobials, which in turn can lead to high mortality rates (Kumar et al., 2009; Gupta et al., 2016). Traditional diagnostic methodologies for septic pathogens encompass the cultivation and isolation of microorganisms, serological detection of pathogen-specific antibodies, antigen identification, and molecular characterization through nucleic acid analysis, predominantly via PCR. Whereas conventional molecular techniques often employ specific primers or probes targeting a restricted array of pathogens, mNGS enable comprehensive characterization of all DNA or RNA within a sample. This approach facilitates a holistic analysis of the entire microbiome and the human host’s genome or transcriptome in clinical specimens (Wensel et al., 2022).

Multiple studies and case reports indicate that genomic DNA or RNA fragments from pathogens involved in infections—whether circulating or non-circulating—can be detected as cell free DNA (cfDNA) or cell-free RNA (cfRNA) in purified plasma (De Vlaminck et al., 2015; Long et al., 2016; Gosiewski et al., 2017; Pan et al., 2017). These findings demonstrate the potential of mNGS for rapid and accurate identification of the pathogens responsible for sepsis (Abril et al., 2016; Hong et al., 2018). Moreover, it can provide detailed information on the abundance of pathogens and their genetic relationships. This technology, therefore, offers significant advantages in diagnosing and understanding the dynamics of infections associated with sepsis (Hong et al., 2018). One study showed that 76% of patients with positive routine blood cultures tested positive for cfDNA mNGS, and only 4% of cfDNA mNGS did not match routine bacterial cultures, and pathogens were accurately determined by cfDNA mNGS combined with analysis of the patient’s clinical presentation in 32.8% of patients with routine blood culture-negative suspected bacteremia (Zhang et al., 2022). This suggests that mNGS can diagnose pathogen infections more accurately than blood cultures. Another study showed that the diagnostic sensitivity was significantly higher than that of blood cultures, providing additional useful information for the development of patient treatment plans (Long et al., 2016). In summary, the advantages in the diagnosis and therapeutic guidance of bloodstream infections are undeniable, as it is effective in reducing the time required for pathogen identification regardless of the microbial type and is less affected by antibiotic administration (Abril et al., 2016). In addition, this method is highly desired for patients infected with rare fungi, mycobacteria and parasites (Miao et al., 2018). In addition, mNGS detects viral infections or mixed infections and guides physicians in the correct and targeted use of antibiotics for septic patients (Hu et al., 2018; Wilson et al., 2018; Xing et al., 2019).

### 4.2 CNS infections

A variety of pathogenic microorganisms can infect the central nervous system, often manifesting as meningitis, encephalitis and abscesses, which may be life-threatening. However, routine microbiological testing is often insufficient to detect all neuroinvasive pathogens, especially rare ones. In addition, obtaining relevant samples for detection of pathogenic pathogens requires invasive procedures such as lumbar puncture or brain biopsy, which are limited by the availability and volume of CSF or brain tissue. As a result, the etiology of CNS infections is often unspecified, which occurs in up to 50 per cent of encephalitis (Glaser et al., 2003; Granerod et al., 2010). Numerous studies have reported the use of mNGS in CSF and brain tissue to detect viruses, bacteria, fungi, and parasites (Xing et al., 2020). In addition, it has proven valuable in diagnosing subacute or chronic meningitis (Wilson et al., 2018). Elevated CSF leukocyte and protein levels, as well as a decreased percentage of glucose in the CSF may be associated with an increase in mNGS detection of CNS infection (Zhang et al., 2020b). A systematic review recommended NGS as a first-line diagnostic test for chronic and recurrent infections and a second-line technique for cases of acute encephalitis (Brown et al., 2018). The detection rate is higher for diagnosing the CNS than traditional pathogen diagnostic methods (Xing et al., 2020; Zhang et al., 2020b). In addition, it may help to rule out active infection in patients with suspected autoimmune encephalitis, providing favourable information for clinicians’ judgement and reducing concerns about missed microbial infections (Wilson et al., 2019; Xing et al., 2020). mNGS results are less affected by the use of antibiotics prior to the collection of CNS samples, and therefore the technique has advantages over other methods for CNS in which antibiotics have been administered (Zhang et al., 2019; Zhang et al., 2020b). Studies have shown that it has high sensitivity, specificity and positive predictive value (PPV) for the diagnosis of CSF tuberculous meningitis (Wang et al., 2019). Indeed, the sensitivity of mNGS was significantly higher than that of culture alone, and the combination of mNGS with conventional methods significantly increased the detection rate. mNGS also has value in identifying complex and rare pathogens present in culture-negative and unconfirmed cases. mNGS has been shown to be useful in detecting the presence of microorganisms such as Listeria monocytogenes, the species that cause brucellosis, *Naegleria fowleri*, the parasites that cause neurocysticercosis, and *Vibrio traumaticus* (Mongkolrattanothai et al., 2017; Fan et al., 2018b; Fan et al., 2018a; Liu et al., 2018; Wang et al., 2018; He et al., 2019). In CNS toxoplasmosis, it can help in the diagnosis when Toxoplasma IgG is negative, CSF PCR is negative, imaging is atypical, or there is a lack of response to anti-Toxoplasma treatment (Hu et al., 2018). In addition, it can be used to dynamically monitor disease progression by analysing semi-quantitative values (Zhang et al., 2020b).

However, mNGS has some shortcomings in diagnosing CNS. While it frequently detects DNA viruses, particularly herpesviruses, its ability to enhance the diagnosis of viral encephalitis and meningitis has not shown significant improvement. One possible reason for this limitation is the underrepresentation of RNA detection methods in current mNGS protocols (Guan et al., 2016; Tyler, 2018; Xia et al., 2019; Fang et al., 2020). Since RNA-based mNGS has not been widely implemented, this restricts the detection of RNA viruses, which are often significant causative agents of these conditions. The lack of comprehensive viral RNA detection may thus hinder the overall effectiveness in diagnosing these serious infections (Xing et al., 2020). Besides, the detection rate of pathogenic microorganisms by mNGS showed a decreasing trend with the prolongation of treatment time (Ai et al., 2018).

### 4.3 Respiratory infections

Upper respiratory tract infections along with lower respiratory tract infections are one of the common diseases and lead to significant mortality (Milucky et al., 2020). Upper respiratory tract infections along with lower respiratory tract infections are one of the common diseases and lead to significant mortality. Undoubtedly, identification and characterization of pathogens is crucial for precise treatment of patients and improved prognosis. However, in the clinical setting, pathogens are often not rapidly identified, which leads physicians to use antibiotics only empirically, which in turn leads to frequent and inappropriate use of antibiotics, and which limits the sensitivity and reliability of culture-based surveillance. mNGS can detect and characterize a wide range of pathogens with relative rapidity and precision, which can contribute to the timely and accurate treatment of lung infections, especially for critically ill patients and patients with mixed infections (Li et al., 2018; van Rijn et al., 2019; Wang et al., 2019; Xie et al., 2019; Huang et al., 2020). In addition, compared with traditional sputum culture or BALF culture, mNGS was able to identify MTB, *nontuberculosis mycobacteria* (NTM), Nocardia, and various Actinomycetes (Miao et al., 2018). For the diagnosis of invasive fungi in the lungs, it is also helpful (Li et al., 2018; Wang et al., 2019). The sensitivity in the diagnosis of mixed lung infections and severe unresponsive pneumonia was evident (Li et al., 2018; Wang et al., 2020). In the analysis of immunocompromised patients, mNGS was even detected with 100% accuracy (Li et al., 2020). Interestingly, it has been reported to be more specific than BALF in transbronchial lung biopsy (TBLB) tissues; however, the BALF assay has been shown to have higher sensitivity in the diagnosis of peripheral lung infectious lesions (Liu et al., 2019). A study showed that for bacteria and fungi, the positive detection rate of mNGS was significantly higher than that of the culture method (91.94% vs 51.61%, P < 0.001), especially for polymicrobial infections (70.97% vs 12.90%, P < 0.001). Compared with the culture method, the diagnostic sensitivity of mNGS was 100%, the specificity was 16.67%, and the PPV and negative predictive value (NPV) were 56.14% and 100%, respectively (Wang et al., 2024). In addition, it more often detects NTM than MTB, Aspergillus or Cryptococcus in BALF (Miao et al., 2018). For human immunodeficiency virus-infected (HIV-infected) patients with suspected lung infections, it can quickly and accurately identify the pathogens that cause lung infections (Hou et al., 2024). Patients with corona virus disease 2019 (COVID-19) may be at increased risk of developing fungal infections as well as concurrent bacterial or viral infections, and mNGS could be a powerful tool for identifying these infections (Huang et al., 2023). Another study showed that BALF mNGS is a valuable tool for differentiating between colonization and infection of *Aspergillu* (Jiang et al., 2024).

### 4.4 Digestive system infection

Although the study of the gut microbiome is a very popular and relevant topic, the use of mNGS technology to diagnose related infectious diseases such as diarrhea has been rarely reported. A case report reporting a definitive diagnosis of *Enterocytozoon bieneusi microsporidiosis* using mNGS suggests that in patients with recurrent unexplained diarrhea with wasting associated with hematological malignancies we should consider the possibility of infection by atypical pathogens. mNGS can help to rule out malignancy and diagnose infection (Zhou et al., 2022). A case report of a definitive diagnosis of *Encephalitozoon hellem* (E. hellem) infection by mNGS in a 9-year-old boy following hematopoietic stem-cell transplantation (HSCT), which led to his timely treatment with albendazole and eventual recovery from reduced immunosuppressive therapy (Shang et al., 2024). A case of Disseminated *Talaromyces marneffei* infection after renal transplantation was also diagnosed and effectively treated by mNGS (Xu et al., 2023). The use of mNGS to assist in the diagnosis and treatment of severe Cryptosporidium infection was reported (Liu et al., 2023; Shan et al., 2023). A patient with diarrhoea was identified as having the causative pathogens MTB complex and *Leptospira* spp. by mNGS of CSF, urine, plasma and sputum clinical samples (Shi et al., 2022). In addition, some cases of *Disseminated histoplasmosis* infection, *Chlamydia psittaci* infection, *Bunyaviridae virus* infection and T. *marneffei* infection diagnosed by mNGS have been reported (Chen et al., 2020; Wang et al., 2022; Zhan et al., 2022; Zheng et al., 2023; Wang et al., 2024). Even, one case reported misdiagnosed tuberculosis being corrected as *Nocardia farcinica* infection by mNGS (Pan et al., 2021). These suggest that mNGS can facilitate diagnosis and timely therapeutic decisions.

### 4.5 Urinary tract infection

UTI is one of the most common infections, affecting 150 million people worldwide each year. The most common cause of UTIs is pathogens (mainly E. coli) in the feces that rise up the urinary tract (Flores-Mireles et al., 2015). Urine culture is the gold standard for the diagnosis of UTI. However, cultures need a long time with low detection rates and limited diagnostic accuracy (Schmiemann et al., 2010). Although PCR methods allow for the rapid detection of pathogens, including non-culturable microorganisms, directly from clinical samples as compared to urine cultures, PCR methods are limited to amplification of predetermined target species and do not meet the diagnostic needs for microorganisms that cannot be predetermined in advance (Smith and Osborn, 2009). The ability of mNGS to detect microorganisms that cause UTIs quickly, accurately, and without prior predetermination allows for the identification of rare, complicated urinary tract infections and is virtually unaffected by prior antibiotic exposure (Miao et al., 2018). A study showed that based on the gold standard of routine culture, mNGS had a sensitivity of 81.4%, a specificity of 92.3%, a PPV of 96.6%, a NPV of 64.9%, and an overall accuracy of 84.4%; while when evaluated based on a composite standard, the sensitivity and the specificity increased to 89.9% and 100%, respectively, and the PPV was 100% and accuracy increased to 92.4% (Wang et al., 2023). Another study shows mNGS-based targeted antibiotic therapy significantly improves urinalysis and urinary symptoms in patients (Jia et al., 2023). Besides, the role of it in the pathogen diagnosis of urinary tract infections in patients after cutaneous ureterostomy, recurrent urinary tract infections in renal transplant recipients, and scrub typhus has been reported (Liu et al., 2021; Duan et al., 2022; Huang et al., 2023). Consequently, mNGS is a technique that offers significant advantages over culture, especially in the case of mixed infections and urinary tract infections that are difficult to diagnose and treat. It helps improve pathogen detection, guides change in treatment strategies, and is a useful complement to urine culture (Jia et al., 2023).

### 4.6 Bone and joint infection

BJI is very serious infection, especially periprosthetic joint infection (PJI), and may even be life-threatening (Malizos, 2017). PJI occurs most often after arthroplasty and multiple revision surgeries. The key to the treatment of PJI is the definitive diagnosis of the causative pathogen. Microbiological cultures are still the primary method for diagnosing PJI, however negative pathogen cultures are a great challenge for clinicians to make treatment decisions (Parvizi et al., 2014; Ahmed and Haddad, 2019). PCR technology has been used to enhance the accuracy of pathogen diagnosis, but it is unable to identify pathogens beyond those that are pre-designed for the disease (Ryu et al., 2014; Hischebeth et al., 2017; Villa et al., 2017). PCR of 16S ribosomal RNA genes has the potential to detect the majority of bacteria but it is not able to identify fungi or multiple microbial infections, nor is it able to distinguish contaminating bacteria from true infecting pathogens (Huang et al., 2018). However, mNGS can provide a comprehensive microbial profile without targeted pre-amplification (Huang et al., 2020). mNGS was reported to be able to identify known pathogens in 94.8% of culture-positive PJI cases and new potential pathogens in 43.9% of culture-negative infections (Thoendel et al., 2018). In one study, in addition to the collection of routine microbiological culture samples, some samples were collected for intraoperative culture to optimize the culture method based on the preoperative mNGS results. Preoperative aspiration of synovial fluid detected by mNGS provides more etiologic information than preoperative cultures, which can guide the optimization of intraoperative cultures and improve the sensitivity of intraoperative cultures (Fang et al., 2021). Another study evaluated the diagnostic value of mNGS using three types of specimens, periprosthetic tissue, synovial fluid, and prosthetic ultrasound-treated fluid, and concluded that it can be used as an accurate diagnostic tool for the detection of pathogens in patients with PJI in all 3 specimens, and that due to its excellence in identifying pathogens, mNGS in artificial ultrasound fluid offers the greatest value and may partially replace traditional tests such as bacterial cultures in these patients (He et al., 2021).

### 4.7 Intra-abdominal infection

Intra-abdominal infection is one of the most common postoperative complications of abdominal surgery. The incidence of postoperative intra-abdominal infection (PIAI) is about 3-10% (Sánchez-Velázquez et al., 2018). Intra-abdominal infections are one of the most common postoperative complications of abdominal surgery. The incidence of postoperative PIAI is about 3-10%. Etiologic evidence remains key to the diagnosis of PIAI to date. However, the diagnosis of causative microorganisms leaves much to be desired (Zhu et al., 2023). First, traditional microbial culture methods are inadequate for isolation of picky microorganisms and anaerobes; second, certain microorganisms are often masked by rapidly proliferating microorganisms, making them difficult to identify; and third, empirical antibiotic treatment prior to sample collection may compromise the sensitivity of culture methods. Finally, traditional methods often take several days to produce results, which may delay appropriate treatment and increase the risk of antibiotic misuse. It was shown that the median sample-to-answer turnaround time for mNGS was significantly lower compared to culture-based methods (<24 h vs. 59.5-111 h). I was detected in a much wider range of assays than culture-based methods (Zhu et al., 2023).

For patients with abdominal sepsis, plasma mNGS can provide early, noninvasive and rapid microbiologic diagnosis. It facilitates rapid detection of pathogenic bacteria compared to traditional peritoneal drainage (PD) smear, culture, and blood culture methods. Paired plasma and PD fluid mNGS improves the microbiologic diagnosis of acute intra-abdominal infections (IAI) compared to microbiologic testing (CMT). The combination of plasma and PD mNGS predicts poor prognosis. It optimizes the use of empiric antibiotics (Li et al., 2022). Patients with spontaneous bacterial peritonitis (SBP) usually receive only empiric antibiotic therapy because pathogens can only be identified in a small number of patients using conventional culture techniques. Compared with conventional culture methods, mNGS improves the detection rate of ascites pathogens (including bacteria, viruses, and fungi), and has significant advantages in diagnosing rare pathogens and pathogens that are difficult to culture; moreover, it may be an effective method to improve the diagnosis of ascites infections in patients with cirrhosis, to guide early antibiotic therapy, and to reduce complications associated with abdominal infections, and relevant cases have been reported (Li et al., 2022; Lei et al., 2023; Shi et al., 2024). Infectious pancreatic necrosis (IPN)-associated pathogens can be identified by plasma mNGS, which has more valuable diagnostic properties and a shorter turnaround time, and may be useful for appropriate treatment (Hong et al., 2022; Lin et al., 2022). mNGS assay improves the detection of pathogenic microorganisms in PD-associated peritonitis, greatly reduces the time to detection, and has good concordance with microbiologic cultures (Zhang et al., 2023). mNGS is advantageous in diagnosing pathogens that are difficult to culture (Ye et al., 2022). mNGS is recommended for patients with PD-associated peritonitis who have received prior antibiotic therapy (Nie et al., 2023).

### 4.8 Skin and soft tissue infection

The diversity of pathogens in skin and soft tissue infections (SSTIs) and the tendency of clinicians to choose broad-spectrum antibiotics increases the prevalence of drug-resistant pathogens, so pathogen identification is important in the rational use of antibiotics (Esposito et al., 2018). Traditional culture techniques are time-consuming and less accurate, and even in many cases the microbial etiology fails to be clearly diagnosed. mNGS has emerged as a technology capable of more accurate diagnosis of pathogens. it has been shown in one study to detect twice as many pathogens compared to traditional culture (67.7% vs. 32.3%), with a particularly high detection rate for anaerobic bacteria, viruses, MTB, and NTM, and was superior in detecting viruses and rare pathogens, even recognizing multiple pathogens in a single specimen (Wang et al., 2020). In addition, positive results significantly contribute to targeted antibiotic therapy and may improve prognosis in the presence of negative culture results. It overcomes the limitations of current diagnostic tests (as 16S rRNA PCR) by allowing universal pathogen detection and new organism detection without *a priori* knowledge of a specific organism. However, in immunocompromised patients, it is less sensitive, possibly due to lower bacterial load (Parize et al., 2017). In conclusion, mNGS can reduce the time to pathogenic diagnosis of SSTI cases and make it possible to give targeted antibiotic therapy in the early stages of infectious diseases.

### 4.9 Intraocular infections

Intraocular infections are often caused by bacteria, fungi, viruses, and parasites, and in some cases can lead to visual impairment or even loss of vision due to poor diagnosis and treatment (Durand, 2017; Dave et al., 2019; Li et al., 2020; Zhang et al., 2020a; Fan et al., 2021). Although intraocular infections have a lower mortality rate compared to other infections, they are still an important cause of blindness (Durand, 2017; Ma et al., 2019). There are a number of cases of intraocular infections that are treated promptly, but the pathogen responsible for the intraocular infection is not diagnosed in time and only antibiotics can be used empirically, eventually leading to worsening of the infection and necrosis of the eye. Instead, only empirical antibiotics can be used, which ultimately leads to worsening of the infection and necrosis of the eyeball, and ultimately the patient’s eyeball is removed (Tsai and Tseng, 2001; Lu et al., 2016). Therefore, timely and accurate diagnosis and treatment have a crucial impact on the prognosis of intraocular infections. The presence of immune privilege in the eye and the blood-ocular barrier makes it difficult to detect intraocular infection pathogens from the blood, so the ideal samples for diagnosing intraocular infection pathogens are vitreous humor (VH) and aqueous humor (AH) (Doan et al., 2016; Deshmukh et al., 2019; Maitray et al., 2019; Li et al., 2020; Fan et al., 2021). The sensitivity of the culture method is only about 40%, and PCR fails to identify pathogens that are not set up for the test (Taravati et al., 2013; Doan et al., 2016; Borroni et al., 2019; Borroni et al., 2022). One study showed that the sensitivity of the mNGS using VH samples had a sensitivity of 92.2% and an overall compliance rate of 81.3%, whereas mNGS using AH samples had a sensitivity of 85.4% and an overall compliance rate of 75.4% (Qian et al., 2024). This indicates that mNGS demonstrates high sensitivity and high overall compliance in the diagnosis of intraocular infections.

## 5 Discussion

mNGS has not been in clinical use for a long time, but with the rapid development of diagnostic molecular microbiology in recent years, it has attracted extensive attention from laboratories and clinics due to its advantages of short time-consumption and wide detection range. Compared with traditional clinical microbiology detection methods, it not only effectively improves the detection rate of pathogenic microorganisms, but also makes up for the shortcomings of traditional detection methods, especially in the precise diagnosis and treatment of certain difficult and serious infectious diseases. So far, although it has been applied in several systemic infections and achieved remarkable results, it has not been widely used in clinical practice because of some shortcomings. Several findings demonstrate the potential benefits of adding it to routine diagnostic workflows for the detection and discovery of rare or novel pathogens, identifying key determinants of clinical benefit, particularly in immunocompromised patients and individuals with brain biopsies or fecal samples (Regnault et al., 2022; Fourgeaud et al., 2023; Pérot et al., 2023; Riller et al., 2023; Fourgeaud et al., 2024).

We think there is a long way to go before mNGS becomes a routine test in the clinic. First, the detection of DNA can only indicate which pathogens are present, not whether they are surviving pathogens. it is also unable to differentiate between pathogenic organisms and one-time bacteremia, so the inclusion of the detection of RNA in mNGS is very necessary. Second, false-negative results are easily produced when the proportion of pathogenic microorganisms in the genus microbacterium is low, so the amounts of precise microorganisms rather than a general determination of species is also in urgent need of improvement. Furthermore, the accuracy can be improved by stipulating uniform procedures and standards to avoid contamination of nucleic acids during specimen collection, transportation, and standardized testing. Finally, the future direction of mNGS is to build a professional team of analysts and interpreters, and to ensure that such a team is trained to a professional and uniform standard, so as to ensure the objectivity and reliability of the result.

Although a great deal of work needs to be improved, we believe that in the next few years, with the development of sequencing technology, the cost and turnaround time of mNGS will continue to decrease, the experimental process will be easy to manipulate, and the whole process will be automated, and it will become an even more mature method of detecting pathogens and play a historic role in clinical diagnosis and treatment.
